# Special Issue: “Advanced Research on Molecular Modeling of Protein Structure and Functions”

**DOI:** 10.3390/ijms26167916

**Published:** 2025-08-16

**Authors:** Maria G. Khrenova

**Affiliations:** 1 Chemistry Department, Lomonosov Moscow State University, 119991 Moscow, Russia; khrenovamg@my.msu.ru; 2 Emanuel Institute of Biochemical Physics of Russan Academy of Sciences, 119334 Moscow, Russia

Recent developments in computer technologies, software and methods have made molecular modeling a powerful tool in experimental studies of biomolecular systems, and in their rational modification. GPU-accelerated molecular dynamics can operate with simulation times of microseconds, allowing researchers to track rare events. Quantum chemical calculations on both CPUs and GPUs can now deal with systems of hundreds of atoms, enabling them to be used in combined quantum mechanics/molecular mechanics biomolecular simulations. These developments have allowed researchers to deepen their understanding of the reaction mechanisms at the active sites of enzymes and photoreceptor proteins, of large conformational rearrangements of protein structures, and of the formation of protein complexes with low-molecular-weight inhibitors or macromolecular compounds. Artificial intelligence-, machine learning- and big data-based analysis methods are becoming an important part of molecular modeling. In this paper, we will present recent advances in molecular modeling related to biomacromolecules.

Significant advances in classical molecular dynamics (MD) simulations driven by improvements in hardware algorithms have revolutionized these methods and expanded the scope of their applications [1]. In the 1990s, small peptides were simulated for several nanoseconds, which was considered as a result significant enough for publication [2]. Later, development of CPU-based supercomputers expanded the range of tasks that can be solved by protein and even protein complex simulations. Still, the trajectory lengths were limited to tens of nanoseconds [3]. Development of specialized supercomputers considerably contributed to developments in this field [4,5]; however, these resources were limited. Emergence of high-performance yet affordable GPUs and further development of software optimized for novel architectures led to a revolution in classical MD simulations. Now, computations of MD trajectories of hundreds of nanoseconds or microseconds for model systems composed of hundreds of thousands of atoms are routine tasks. This allows researchers to track large conformational changes and existence of different states of entire proteins.

Development of enhanced sampling methods has further extended the possible applications of MD methods. Many of them require explicit definitions of so-called collective variables, which are usually functions of structural parameters, for example, their linear combinations [6,7]. The most frequently used methods are metadynamics and umbrella sampling. Umbrella sampling presumes addition of an external potential, usually harmonic, centered at a certain value of the collective variable, and analyzes the equilibrium distribution of states [8,9,10]. In contrast, metadynamics utilizes nonequilibrium sampling, allowing researchers to reconstruct an equilibrium free-energy landscape [11]. Both of these methods are frequently utilized to describe transitions between different states, particularly those actively applied to study binding processes [12,13,14,15].

Large-scale MD simulations produce gigabytes of atomic coordinates and thousands of trajectory frames that cannot be analyzed by visual inspection and evaluation of simple geometry parameters (interatomic distances, hydrogen bonds, etc.) or using cumulative descriptors like RMSD and RMSF [16]. Valuable information can be extracted from MD trajectories using modern methods such as big data analysis and machine learning [16,17,18,19,20,21,22,23]. Kinetic analysis of transitions between different states is usually studied using Markov state modeling (MSM), which utilizes distributed MD simulation data to determine long-term behavior [24,25]. This approach requires so-called featurization and dimension reduction to a set of slow collective variables [26,27]. Recently, the variational approach for Markov processes (VAMP) was proposed to develop a deep learning framework for molecular kinetics using neural networks, called VAMPnet [28,29]. It combines the whole data processing pipeline into a single end-to-end framework and encodes the entire mapping from molecular coordinates to Markov states, providing easily interpretable kinetic models with few states.

Data clustering is frequently applied to large MD trajectories and obtains ensembles with different conformations [16]; MDTraj [30] and EnGens [31] allow researchers to perform this analysis. Interestingly, analyzing sets of static geometry parameters and their changes along trajectories can enable researchers to distinguish fragments of large biomolecular systems that are characterized by correlated motions, making this a powerful method for characterizing interactions in enzyme–inhibitor complexes [32,33,34].

QM/MM methods are mostly used to study chemical and photochemical/photophysical processes in biomacromolecular systems [35,36,37]. Initially, QM/MM simulations were utilized to locate stationary points on potential energy surfaces (PESs) and reconstruct reaction mechanisms. Today, QM/MM is also utilized for molecular dynamics simulations [38,39,40,41,42,43,44,45]. This considerably improves out understanding and provides a more realistic description of reaction mechanisms as proteins and their active sites have numerous conformations. Therefore, dealing with a particular PES minimum can lead to an incorrect qualitative understanding of the reaction mechanism. For example, a high-energy minimum can be practically unpopulated but serves as an initial state of energetically favorable reaction pathway. Different studies have demonstrated the existence of reactive and non-reactive reagent states that can only be tracked in molecular dynamics simulations [46,47].

Recent years have seen the emergence of an interdisciplinary field combining artificial intelligence (AI), machine learning (ML) and molecular modeling. Many of these methods are based on sequence inputs and utilize complex algorithms trained on large databases for predictions of 3D structures [48,49] and required properties [50].

Computer and software developments in the last decade have considerably increased the power of molecular modeling and its application in solving a wide variety of tasks. Big data analysis further enhances the potential of molecular modeling due to its ability to extract structural and dynamic data that cannot be obtained through visual analysis. Utilization of AI and ML algorithms in molecular modeling represents the next step in further developing this field of research.
